# Prevalence and Clinical Characteristics of the *LRRK2* p.L1795F Variant in Central Europeans with Early‐Onset and Familial Parkinson's Disease

**DOI:** 10.1002/mdc3.70045

**Published:** 2025-03-22

**Authors:** Miriam Ostrozovicova, Gertrud Tamas, Agsha Atputhavadivel, Petr Dusek, Milan Grofik, Vladimir Han, Petr Holly, Robert Jech, Katarina Kalinova, Peter Klivenyi, Norbert Kovacs, Kristina Kulcsarova, Egon Kurca, Alexandra Lackova, Hamin Lee, Patrick Lewis, Veronika Magocova, Maria Marekova, David Murphy, Ai Nagano, Jan Necpal, David Pinter, Miroslava Rabajdova, Evzen Ruzicka, Tereza Serranova, Katarzyna Smilowska, Krisztina Soos, Igor Straka, Tatiana Svorenova, Peter Valkovic, Katerina Zarubova, Zuzana Gdovinova, Henry Houlden, Mie Rizig, Matej Skorvanek, the CEGEMOD consortium, Dusek Petr, Holly Petr, Jech Robert, Ruzicka Evzen, Serranova Tereza, Zarubova Katerina, Klivenyi Peter, Kovacs Norbert, Pinter David, Tamas Gertrud, Smilowska Katarzyna, Grofik Milan, Han Vladimir, Kulcsarova Kristina, Kurca Egon, Lackova Alexandra, Magocova Veronika, Necpal Jan, Ostrozovicova Miriam, Skorvanek Matej, Straka Igor, Svorenova Tatiana, Valkovic Peter, Houlden Henry, Rizig Mie

**Affiliations:** ^1^ Department of Neurology P.J. Safarik University Kosice Slovak Republic; ^2^ Department of Neurology University Hospital of L. Pasteur Kosice Slovak Republic; ^3^ Department of Neuromuscular Diseases UCL Queen Square Institute of Neurology London United Kingdom; ^4^ Department of Neurology Semmelweis University Budapest Hungary; ^5^ Department of Neurology and Centre of Clinical Neuroscience First Faculty of Medicine, Charles University and General University Hospital in Prague Prague Czech Republic; ^6^ Department of Neurology Jessenius Faculty of Medicine, Comenius University and University Hospital Martin Martin Slovak Republic; ^7^ Division of Molecular Biology and Biochemistry Gottfried Schatz Research Center, Medical University of Graz Graz Austria; ^8^ Department of Neurology University of Szeged Szeged Hungary; ^9^ Department of Neurology and HUN‐REN–PTE Clinical Neuroscience MR Research Group University of Pecs, Medical School Pécs Hungary; ^10^ Department of Clinical Neurosciences Scientific Park MEDIPARK, P.J. Safarik University Kosice Slovak Republic; ^11^ Royal Veterinary College London United Kingdom; ^12^ Department of Neurodegenerative Disease UCL Queen Square Institute of Neurology London United Kingdom; ^13^ Department of Medical and Clinical Biochemistry Faculty of Medicine, P.J. Safarik University in Kosice Kosice Slovak Republic; ^14^ Department of Clinical and Movement Neurosciences UCL Queen Square Institute of Neurology, University College London London United Kingdom; ^15^ Department of Neurology Zvolen Hospital Zvolen Slovak Republic; ^16^ Department of Neurology Silesian Centre of Neurology Katowice Katowice Poland; ^17^ Second Department of Neurology Comenius University in Bratislava Faculty of Medicine, University Hospital Bratislava Bratislava Slovak Republic; ^18^ Institute of Normal and Pathological Physiology, Centre of Experimental Medicine Slovak Academy of Sciences Bratislava Slovak Republic; ^19^ Department of Neurology, Second Faculty of Medicine Charles University and Motol University Hospital Prague Czech Republic

**Keywords:** leucine‐rich repeat kinase 2 (LRRK2), L1795F, Parkinson's disease, risk factor, mutation, genetics

## Abstract

**Background:**

Leucine‐rich repeat kinase 2 (*LRRK2*) p.L1795F variant was proposed as a genetic risk factor for Parkinson's disease (PD). However, its prevalence, phenotype, and origin remain unknown.

**Objective:**

The aim was to evaluate the frequency and phenotype of p.L1795F in early‐onset PD (EOPD) and familial PD compared to healthy controls (HC) in Central Europe.

**Methods:**

Whole‐exome sequencing was used to screen 219 EOPD and familial PD patients of Central Europeans compared to HC. Sanger sequencing assessed segregation. Detailed clinical phenotype was evaluated for all positive carriers.

**Results:**

p.L1795F was identified in 1.37% (3/219) and 3.23% of familial cases (3/93), with no carriers among HCs (0/303). Segregation analysis confirmed association with PD. Carriers were traced to the eastern Slovak–Hungarian region. It also appears to be associated with a more aggressive phenotype.

**Conclusion:**

Our data indicate that p.L1795F contributes to PD in Central Europe. Further exploration in larger cohorts is warranted to establish its contribution to global PD risk.

Recently, the p.L1795F (rs111910483, c.5385G>T) variant was proposed as a genetic risk factor for Parkinson's disease (PD).^1^ It was also previously shown to exert a functional effect via enhanced kinase activity, providing more evidence for its pathogenicity.^2^ The p.L1795F variant was previously identified in 2 PD patients—siblings within a family with several family members affected. No segregation was shown due to the unavailability of additional family members.^3^ It was also reported in a single PD case (1/478) of European‐American ancestry.^4^ However, further reports are currently lacking in the literature.

We previously reported that the globally observed leucine‐rich repeat kinase 2 (*LRRK2*) pathogenic variants, such as p.G2019S, are infrequent in PD patients of Central European ancestries.^5^ This study seeks to build upon our prior discoveries by examining the newly suggested p.L1795F variant and its related clinical phenotype in early‐onset PD (EOPD) and (or) familial cases versus healthy controls (HC) in Central Europeans.

## Patients and Methods

Patients with EOPD and/or familial PD (n = 219) and geographically matched HCs (n = 303) were recruited from 9 movement disorder centers in the Czech Republic, Hungary, Poland, and Slovakia within the CEGEMOD consortium as described previously.^6^ The research protocol was approved by the ethics committees from all participating centers. All patients provided informed consent. Each individual with PD was diagnosed in accordance with the Movement Disorder Society (MDS) clinical diagnostic criteria.^7^ Recruitment and clinical assessments are presented in the Supplementary Materials. The genetic analysis included DNA extraction and whole‐exome sequencing (WES) of PD cases, whereas the Competitive Allele Specific PCR (KASP) assay screened geographically matched HCs. Segregation analysis was performed using Sanger sequencing in identified families. For all 4 index PD patients, genotyping data were obtained for haplotype analysis and mutation dating. Structural modeling was derived from Protein Data Bank 7LHT structure (PDB 7LHT).^8^ Detailed methodology of all genetic studies is presented the Supplementary Materials and in Table S1.

## Results

Our study included 219 patients with EOPD and/or family history of PD from 4 Central European countries of the Visegrad group: the Czech Republic, Hungary, Poland, and Slovakia. A positive family history was reported in 93 patients (42%), and 117 patients (53%) developed PD before the age of 40 years. The average age of PD patients was 53.5 ± 12.9 years, with 136 (62%) being men. In addition, 303 geographically matched HCs were screened in this study to assess the p.L1795F variant's frequency within the studied population. The demographic characteristics of the cohort are provided in Tables S2 and S3.

**FIG. 1:**
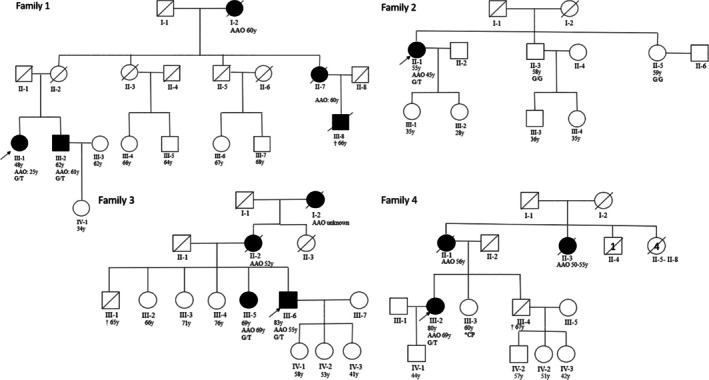
Pedigrees of *LRRK2* (leucine‐rich repeat kinase 2) p.L1795F‐positive PD patients. AAO, age at onset; CP, cerebral palsy; G/G, homozygous for the wild‐type G allele; G/T, heterozygous p.L1795F variant carrier; y, years.

We identified 4 PD cases carrying the heterozygous *LRRK2* p.L1795F variant. Three carriers (F1‐III‐1, previously reported elsewhere^9^; F3‐III‐6 and F4‐III‐2) were discovered through the original WES study group, and 1 additional carrier (F2‐II‐1) was later included based on positive clinical genetic report (Table S4). No other pathogenic variants in PD‐related genes were identified in the 3 PD cases with WES data available (Table S4). Similarly, genotyping data of all 4 index PD patients excluded pathogenic copy number variants in PD‐related genes (Fig. S1). The age of onset (AAO) was 25, 45, 55, and 69, with a mean AAO of 48.5 ± 18.5 years. Interestingly, the patient with the youngest AAO at 25 years (F1‐III‐1) also carried rare heterozygous *MAPT* p.R538P (c.1613G>C) variant with unknown clinical significance (*CADD score* 25.1; *polyphen*: probably damaging; *SIFT*: deleterious, *carol*: deleterious) as no reports are available in the literature. Three cases had a positive family history (75%), with several family members affected (Table 1). Sanger sequencing was used in all relatives with DNA available for the segregation analysis, showing that p.L1795F variant segregated with PD phenotype (Fig. 1; Fig. S2). Interestingly, all patients were from the same region close to the east Slovak–Hungarian border (Tokaj region) (Table 1). None of the 4 index cases were distant relatives (Table S5). The estimated frequency of the p.L1795F variant in the original EOPD and familial PD cohort is 1.37% (3/219) and 3.23% (3/93) in the familial cases. Of note, no other *LRRK2* variants' carriers were identified in this cohort except for a single EOPD case with the heterozygous p.N1437S variant. All geographically matched HCs were negative for p.L1795F variant.

**TABLE 1:** *Demographics and clinical characteristics of identified* LRRK2 *p.L1795F‐positive PD patients*

Patient ID	F1‐III‐1	F1‐III‐2	F2‐II‐1	F3‐III‐5	F3‐III‐6	F4‐III‐2
Gender	F	M	F	F	M	F
Origin	Hungarian	Hungarian	Slovak	Slovak	Slovak	Slovak
Age (y)	48	62	55	69	83	80
Age at onset (y)	25	61	45	60	55	69
Disease duration (y)	23	1	10	9	28	11
Family history of PD	Positive	Positive	Negative	Positive	Positive	Positive
Family members affected with PD	Brother (F1‐III‐2), maternal aunt and grandmother	Sister (F1‐III‐1), maternal aunt and grandmother	None	Brother (F3‐III‐5), mother, maternal grandmother	Sister (F3‐III‐5), mother, maternal grandmother	Mother, mother's sister
PD subtype	Akinetic rigid	Mixed	Akinetic rigid	Akinetic rigid	Akinetic rigid	Akinetic rigid
Initial motor features	Unilateral bradykinesia and rigidity	Unilateral bradykinesia, rigidity, and resting tremor	Unilateral bradykinesia and rigidity	Unilateral bradykinesia and rigidity	Unilateral bradykinesia and rigidity	Unilateral bradykinesia and rigidity
MDS‐UPDRS	4 on	10 on	14 on	30 on	58 on	29 on
Part III on/off	48 off	NA	31 off	NA	NA	NA
Bradykinesia	+	+	+	+	+	+
Rigidity	+	+	+	+	+	+
Resting tremor	−	+	−	−	−	−
Freezing	+	−	−	−	+	−
Postural instability	+	−	+	+	+	+
Dyskinesia	+	−	+	+	+	+
H&Y stage	3	1	3	3	5	3
Early motor fluctuations	+	−	+	+	+	+
MoCA score	29	25	26	NA	13	23
Neuropsychiatric features (self‐reported)	Depression	Depression, anxiety, apathy	Depression, anxiety, apathy	None	None	Depression, anxiety
Nonmotor features	Fatigue, nocturia, constipation, light headedness on standing	Fatigue, heat/cold intolerance, postural hypotension	Fatigue, insomnia, urinary urgency, constipation, light headedness on standing, excessive sweating, chronic pain	Constipation, light headedness on standing	Fatigue, nocturia, urinary urgency, light headedness on standing, chronic pain	Fatigue, insomnia, urinary urgency, light headedness on standing, chronic pain
Other features	Hypercholesterolemia, endometriosis, hydronephrosis caused by ureteric stones	Benign prostate hyperplasia, chronic back pain (disk prolapse)	Hypothyroidism	Osteoporosis, hypothyroidism, arterial hypertension	Osteoarthrosis	Arterial hypertension, hypercholesterolemia, osteoarthrosis
Response to levodopa	+	Not tried	+	+	+	+
Current medication	STN DBS, l‐dopa/carbidopa/entacapone (300/75/1200 mg/day) Pramipexole (1.04 mg/day) Amantadine (300 mg/day)	Rasagiline (1 mg/day)	STN DBS, l‐dopa/carbidopa (250/25 mg/day), amantadin (100 mg/day) Rasagiline (1 mg/day) Opicapone (50 mg/day)	l‐Dopa/carbidopa (425/106.25 mg/day), opicapone (50 mg/day) Ropinirole (2 mg/day) Amantadine (300 mg/day) Rasagiline (1 mg/day)	LCIG (6.7 ml/hour) Amantadin (300 mg/day) Opicapone (50 mg/day)	LCIG (6.7 ml/hour) l‐Dopa/carbidopa (100/25 mg/day)
Therapeutical effect on motor fluctuations	Unsatisfactory	Satisfactory	Unsatisfactory	Unsatisfactory	Unsatisfactory	Unsatisfactory

Abbreviations: *LRRK2*, leucine‐rich repeat kinase 2; PD, Parkinson's disease; MDS‐UPDRS, Movement Disorder Society‐Unified Parkinsons Disease Rating Scale; NA, not available; H&Y, Hoehn and Yahr; MoCA, Montreal Cognitive Assessment; STN, subthalamic nucleus; DBS, deep brain stimulation; LCIG, levodopa‐carbidopa intestinal gel; l‐dopa, levodopa.

Clinical features of PD patients (n = 6) carrying the p.L1795F variant are presented in Table 1 and Table S6. Of all identified cases, 5 were characterized as akinetic‐rigid PD subtype, responsive to levodopa treatment in early stages. One PD case presented as mixed PD phenotype with mild resting tremor, bradykinesia, and rigidity. Postural instability later developed in 5 cases (84%), with freezing being present in 2 cases (34%). Detailed clinical phenotype is described in the Supplementary Materials.

Identity by Descent (IBD) analyses in patients F1‐III‐1, F3‐III‐6, and F4‐III‐2 first identified a shared segment of a median size of ≈10 cM (Table S7). Additionally, the IBD analysis of the array dataset available from the fourth patient (F2‐II‐1) in combination with 3 other patients revealed a 2‐cM shared segment, consistently detected by both Hap‐IBD and Germline2. All 4 carriers shared core haplotype spanning ≈2 kbp at this locus (Table S8), suggesting that the p.L1795F variant originated from a common ancestor. We estimated the age of the p.L1795F variant in our cohort to be between 285 and 2369 years, with a 95% confidence interval. The CryoEM structure revealed that p.L1795F residue is situated near pathogenic mutations in the Ras of complex proteins (ROC) and the C‐terminal of ROC (COR) domains (Fig. S3). Previous biochemical studies of p.L1795F variant indicated an approximately 5‐fold increase in Rab10 phosphorylation at p.T73, well‐established *LRRK2* substrate, compared to the wild‐type protein.^2^ Additionally, there was an approximate 2‐fold increase in autophosphorylation at p.S1292 and a reduction by half in phosphorylation at p.S935.^2^

## Discussion

Recently, the *LRRK2* p.L1795F variant has been suggested as a possible genetic risk factor for PD.^1^ This study evaluated the potential contribution of the p.L1795F variant to PD in the Central European countries of the Visegrad group, a region with a largely uncharted PD genetic landscape compared to other European populations due to limited genetic studies. Our analysis concentrated on a cohort of PD patients with EOPD and/or positive family history (Table S2).

We initially identified 3 p.L1795F heterozygous PD carriers out of 219 EOPD and/or familial PD cases (Table 1). One PD case was additionally identified via a clinical genetic report of PD panel testing conducted previously (Table S4). The frequency of the p.L1795F variant was 1.37% (3/219) and 3.23% (3/93) in familial PD cases, significantly higher than the reported prevalence of the known pathogenic *LRRK2* variants like p.G2019S, which is estimated to have a prevalence of about 0.33%.^5^ It is well recognized that *LRRK2* variants show population diversity, with prevalence differing across regions and ancestries.^10^ Interestingly, all identified PD p.L1795F carriers were unrelated (Table S5) but could be traced back to the same east Slovak–Hungarian (Tokaj) region. In addition, they shared common haplotype segment, suggesting common ancestor (Tables S7 and S8). We estimated that the founding event of this variant occurred ~285 to 2369 years ago. Several observations support pathogenicity of p.L1795F in Europeans: the p.L1795F variant is extremely rare in population databases and appears to be confined to individuals of European ancestry. In the *gnomAD*, version 4.1, database, only 2 of 1,613,650 individuals were identified as carriers.^11^ In the PD variant browser, only 4 PD patients of 5811 cases were identified, with no carriers found among the 6207 HCs.^12^ Similarly, none of the geographically matched HCs within our study carried the variant (0/303). Additionally, p.L1795F was identified in several unrelated individuals with the same phenotype, both within our cohort and prior studies.^13^ The *LRRK2* p.L1795F variant co‐segregated with PD in families where several members could be examined (Families 1, 2, and 3: Fig. 1; Fig. S2). Although parents and additional relatives were not available for genetic testing, a positive family history of PD across at least 2 generations in these carriers (see pedigrees: Fig. 1) suggests Mendelian inheritance. Previous in vitro functional analyses demonstrated that p.L1795F can enhance LRRK2 kinase activity and was computationally predicted as likely pathogenic or damaging (AlphaMissense: likely pathogenic with a score of 0.744,^14^ REVEL score = 0.638, conservation score = 9).^2^ These data, along with our in silico modeling (Fig. S3), suggest that p.L1795F has a functional impact similar to that observed in pathogenic mutations in the ROC and COR domains. The p.L1795F phenotype appears to consist of *LRRK2*‐associated PD, resembling idiopathic PD.^15^ The AAO varies from early (25 years) to late (69 years) age (Table 1), as in the first family reported previously with 2 siblings diagnosed as late‐onset PD (60 and 66 years).^3^ The patient with the AAO at 25 years also carried a rare heterozygous *MAPT* p.R538P (c.1613G>C; p.Arg538Pro). Several polymorphic *MAPT* variations in the *MAPT* gene have already been shown to possibly influence the AAO in *LRRK2‐*associated PD,^16^, ^17^ though results are inconclusive and mechanism remains unknown. Interestingly, the majority (83%) of the identified cases in our study lack tremor in clinical presentation, as well as have very early onset of severe dyskinesia and motor fluctuations, with a narrow therapeutic window and unsatisfactory response to advanced treatment options (levodopa–carbidopa intestinal gel or deep brain stimulation) in 4 cases. Only 1 PD case reported mild resting tremor and did not report any motor fluctuations, though the disease duration was only 1 year. Orthostatic hypotension (83%) and urinary dysfunction (83%) were reported as the most common nonmotor symptoms. No PD case was diagnosed with rapid eye movement sleep behavior disorder, and only 1 patient was diagnosed with level 1 PD dementia based on the Montreal Cognitive Assessment score (13 points), being in his early eighties and after 28 years of diagnosis. Neuropsychiatric features, such as anxiety or depression, were also self‐reported in 4 cases (67%).

In summary, the genetic analysis, combined with the segregation analysis, structural data, and the variant's frequency in patients compared to geographically matched HCs and publicly available datasets with previously published studies, suggests a potential pathogenic role for the p.L1795F variant in PD. Consequently, we recommend including this variant in standard genetic testing for PD patients in Central Europe, as it appears to contribute to PD with a possible common ancestor from this region. Further screening in large PD cohorts and additional functional studies, such as assessing kinase activities in cell lines, are necessary to fully understand its role in PD and the full phenotypic spectrum. Alongside ongoing clinical trials for LRRK2 inhibitors, this finding highlights the urgent need for greater ethnic diversity in PD genetic research.

## Author Roles

(1) Research project: A. Conception, B. Organization, C. Execution; (2) Statistical analysis: A. Design, B. Execution, C. Review and critique; (3) Manuscript: A. Writing of the first draft, B. Review and critique.

M.O.: 1A, 1B, 1C, 2A, 2B, 3A

G.T.: 1A, 1B, 1C, 3B

A.A.: 2A, 2B

P.D.: 1B, 1C, 2C, 3B

M.G.: 1B, 1C, 2C, 3B

V.H.: 1B, 1C, 2C, 3B

P.H.: 1B, 1C, 2C, 3B

R.J.: 1B, 1C, 2C, 3B

K.K.: 1B, 1C, 2A, 2B, 2C, 3B

P.K.: 1B, 1C, 2C, 3B

N.K.: 1B, 1C, 2C, 3B

E.K.: 1B, 1C, 2C, 3B

A.L.: 1B, 1C, 2C, 3B

H.L.: 2A, 2B

P.L.: 2A, 2B, 2C, 3B

V.M.: 1C, 3B

M.M.: 1C, 3B

D.M.: 2A, 2B, 2C, 3B

A.N.: 2A, 2B, 2C, 3B

J.N.: 1A, 1B, 1C, 3B

D.P.: 1A, 1B, 1C, 3B

M.R.: 1A, 1B, 1C, 2C, 3B

E.R.: 1A, 1B, 1C, 3B

T.S.: 1A, 1B, 1C, 3B

K.Sm.: 1A, 1B, 1C, 3B

K.So.: 1A, 1B, 1C, 3B

I.S.: 1A, 1B, 1C, 3B

P.V.: 1A, 1B, 1C, 3B

K.Z.: 1B, 1C, 3B

Z.G.: 1A, 1B, 3B

H.H.: 1A, 1B, 2C, 3B

M.S.: 1A, 1B, 1C, 2C, 3B

## Disclosures

**Ethical Compliance Statement**: This study was approved by the University Hospital of L. Pasteur Research Ethics Board. Written informed patient consent was obtained from each participant. We confirm that we have read the journal's position on issues involved in ethical publication and affirm that this work is consistent with those guidelines.

**Funding Sources and Conflicts of Interest**: This study was funded by the Slovak Grant and Development Agency under contracts APVV‐22‐0279, by the Slovak Scientific Grant Agency under contract VEGA 1/0712/22, and by the EU Renewal and Resilience Plan “Large Projects for Excellent Researchers” under grant number 09I03‐03‐V03‐00007. The Czech center was supported by project number LX22NPO5107 (MEYS): financed by the European Union–Next Generation EU and by the Czech Health Research Council grant NU21‐04‐00535 and MH CZ‐DRO‐VFN64165. P.K. is supported by a TKP2021‐EGA‐32 grant that has been implemented with the support provided by the Ministry of Culture and Innovation of Hungary from the National Research, Development and Innovation Fund, financed under the TKP2021‐EGA funding scheme. P.K.'s work also contributed to the Rare Neurological Disorders‐European Reference Network. P.L. is a Royal Society Industry Research Fellow (IF\R2\222002). The authors declare that there are no conflicts of interest relevant to this work.

**Financial Disclosures for the Previous 12 Months**: The authors declare that there are no additional disclosures to report.

## Supporting information


**Table S1.** Primer design and optimised PCR programme used for p.L1795F variant validation.
**Table S2.** Characteristics of the PD patients included in the WES study group.
**Table S3.** Characteristics of the HC included in the study.
**Table S4.** List of PD‐associated genes screened in our PD cohort.
**Table S5.** Identity‐by‐descent (IBD) calculation.
**Table S6.** Additional clinical information of identified LRRK2 p.L1795F positive PD patients.
**Table S7.** The overlapping identify‐by‐descent segments spanning LRRK2 p.L1795F variant among the carriers genotyped by whole‐exome sequence and array.
**Table S8.** The common haplotype (grey) shared by LRRK2 p.L1795F (red) carriers inferred from the whole‐exome‐sequence and array data.
**Figure S1.** B‐allele frequency and Log‐R ratio plots of the LRRK2 p.L1795F positive carriers.
**Figure S2.** p.L1795F variant's validation by Sanger sequencing.
**Figure S3.** (A) CryoEM structure for the LRRK2 dimer with highlighted PD‐associated mutations including the proposed p.L1795F variant (B) proximity of p.L1795F to previously demonstrated pathogenic variants in the ROC and COR domains. Image derived from PDB 7LHT using chimera X.

## Data Availability

The data that support the findings of this study are available from the corresponding author upon reasonable request.

## Appendix A

### A.1 MEMBERS OF THE CENTRAL EUROPEAN GROUP ON GENETICS OF MOVEMENT DISORDERS (CEGEMOD)

#### A.1.1 MEMBERS BY COUNTRY

##### A.1.1.1 Czech Republic

Dusek Petr, Department of Neurology and Centre of Clinical Neuroscience, First Faculty of Medicine, Charles University and General University Hospital in Prague, Prague, Czech Republic. petr.dusek@vfn.cz.

Holly Petr, Department of Neurology and Centre of Clinical Neuroscience, First Faculty of Medicine, Charles University and General University Hospital in Prague, Prague, Czech Republic. petr.holly@vfn.cz.

Jech Robert, Department of Neurology and Centre of Clinical Neuroscience, First Faculty of Medicine, Charles University and General University Hospital in Prague, Prague, Czech Republic. jech@cesnet.cz.

Ruzicka Evzen, Department of Neurology and Centre of Clinical Neuroscience, First Faculty of Medicine, Charles University and General University Hospital in Prague, Prague, Czech Republic. evzen.ruzicka@lf1.cuni.cz.

Serranova Tereza, Department of Neurology and Centre of Clinical Neuroscience, First Faculty of Medicine, Charles University and General University Hospital in Prague, Prague, Czech Republic. tereza.serranova@lf1.cuni.cz.

Zarubova Katerina, Department of Neurology, Second Faculty of Medicine, Charles University and Motol University Hospital, Prague, Prague, Czech Republic. katzar@centrum.cz.

##### A.1.1.2 Hungary

Klivenyi Peter, Department of Neurology, University of Szeged, Szeged, Hungary. klivenyi.peter@med.u-szeged.hu.

Kovacs Norbert, University of Pecs, Medical School, Department of Neurology and HUN‐REN–PTE Clinical Neuroscience MR Research Group. kovacsnorbert06@gmail.com.

Pinter David, University of Pecs, Medical School, Department of Neurology and HUN‐REN–PTE Clinical Neuroscience MR Research Group. david_pinter@outlook.com.

Tamas Gertrud, Department of Neurology, Semmelweis University, Budapest, Hungary. tamas.gertrud@med.semmelweis-univ.hu.

##### A.1.1.3 Poland

Smilowska Katarzyna, Department of Neurology, Silesian Centre of Neurology, Katowice, Poland. kasia.smilowska@gmail.com.

##### A.1.1.4 Slovak Republic

Grofik Milan, Department of Neurology, Commenius University and University Hospital Martin, Martin, Slovak Republic. milangrofik@gmail.com.

Han Vladimir, Department of Neurology, Safarik University and L. Pasteur University Hospital Kosice, Slovak Republic. vladimir.han@gmail.com.

Kulcsarova Kristina, Department of Neurology, Safarik University and L. Pasteur University Hospital Kosice, Slovak Republic. kristina.kulcsarova@upjs.sk.

Kurca Egon, Department of Neurology, Commenius University and University Hospital Martin, Martin, Slovak Republic. egonkurca@gmail.com.

Lackova Alexandra, Department of Neurology, Safarik University and L. Pasteur University Hospital Kosice, Slovak Republic. alexandra.mosejova@gmail.com.

Magocova Veronika, Department of Neurology, Safarik University and L. Pasteur University Hospital Kosice, Slovak Republic. veronikamagocova.a@gmail.com.

Necpal Jan, Department of Neurology, Zvolen Hospital, Zvolen, Slovak Republic. necpal.neuro@gmail.com.

Ostrozovicova Miriam, Department of Neurology, Safarik University and L. Pasteur University Hospital Kosice, Slovak Republic. miriam.ostrozovicova@gmail.com; m.ostrozovicova@ucl.ac.uk.

Skorvanek Matej, Department of Neurology, Safarik University and L. Pasteur University Hospital Kosice, Slovak Republic. mskorvanek@gmail.com.

Straka Igor, Second Department of Neurology, Comenius University in Bratislava Faculty of Medicine, University Hospital Bratislava, Bratislava, Slovak Republic. straka0105@gmail.com.

Svorenova Tatiana, Department of Neurology, Safarik University and L. Pasteur University Hospital Kosice, Slovak Republic. talorinc@gmail.com.

Valkovic Peter, Second Department of Neurology, Comenius University in Bratislava Faculty of Medicine, University Hospital Bratislava, Bratislava, Slovak Republic; Institute of Normal and Pathological Physiology, Centre of Experimental Medicine, Slovak Academy of Sciences, Bratislava, Slovak Republic. peter.valkovic@gmail.com.

##### A.1.1.5 United Kingdom

Houlden Henry, Department of Neuromuscular Diseases, UCL Queen Square Institute of Neurology, London, United Kingdom. h.houlden@ucl.ac.uk.

Rizig Mie, Department of Neuromuscular Diseases, UCL Queen Square Institute of Neurology, London, United Kingdom. m.rizig@ucl.ac.uk.

## References

[1] Pitz V , Makarious MB , Bandrés‐Ciga S , et al. Analysis of rare Parkinson's disease variants in millions of people. Res Sq 2024;10(1):11. 10.1038/s41531-023-00608-8. PMC10774311 38191580

[2] Kalogeropulou AF , Purlyte E , Tonelli F , et al. Impact of 100 LRRK2 variants linked to Parkinson's disease on kinase activity and microtubule binding. Biochem J 2022;479:1759–1783. 35950872 10.1042/BCJ20220161 PMC9472821

[3] Nichols WC , Elsaesser VE , Pankratz N , et al. LRRK2 mutation analysis in Parkinson disease families with evidence of linkage to PARK8. Neurology 2007;69(18):1737–1744. 10.1212/01.wnl.0000278115.50741.4e. 17804834

[4] Benitez BA , Davis AA , Jin SC , et al. Resequencing analysis of five mendelian genes and the top genes from genome‐wide association studies in Parkinson's disease. Mol Neurodegener 2016;11:29. 27094865 10.1186/s13024-016-0097-0 PMC4837564

[5] Skorvanek M , Rizig M , Athanasiou‐Fragkouli A , et al. LRRK2 mutations in Parkinson's disease patients from Central Europe: a case control study. Parkinsonism Relat Disord 2021;83:110–112. 33561776 10.1016/j.parkreldis.2020.12.021

[6] Ostrozovicova M , Dusek P , Grofik M , et al. Central European group on Genetics of movement Disorders. Eur J Neurol 2024;31:e16165. 38059386 10.1111/ene.16165 PMC11236003

[7] Postuma RB , Berg D , Stern M , et al. MDS clinical diagnostic criteria for Parkinson's disease. Mov Disord 2015;30:1591–1601. 26474316 10.1002/mds.26424

[8] Myasnikov A , Zhu H , Hixson P , et al. Structural analysis of the full‐length human LRRK2. Cell 2021;184:3519–3527.e10. 34107286 10.1016/j.cell.2021.05.004 PMC8887629

[9] Illés A , Csabán D , Grosz Z , et al. The Role of Genetic Testing in the Clinical Practice and Research of Early‐Onset Parkinsonian Disorders in a Hungarian Cohort: Increasing Challenge in Genetic Counselling, Improving Chances in Stratification for Clinical Trials. Front Genet 2019;10:1061. 10.3389/fgene.2019.01061. 31737044 PMC6837163

[10] Simpson C , Vinikoor‐Imler L , Nassan FL , Shirvan J , Lally C , Dam T , Maserejian N . Prevalence of ten LRRK2 variants in Parkinson's disease: a comprehensive review. Parkinsonism Relat Disord 2022;98:103–113. 35654702 10.1016/j.parkreldis.2022.05.012

[11] Chao K . GnomAD v4.0; https://gnomad.broadinstitute.org/news/2023-11-gnomad-v4-0/.

[12] No title . https://pdgenetics.shinyapps.io/VariantBrowser.

[13] Pitz V , Makarious MB , Bandres‐Ciga S , et al. Analysis of rare Parkinson's disease variants in millions of people. NPJ Parkinsons Dis 2024;10:1–10. 38191580 10.1038/s41531-023-00608-8 PMC10774311

[14] Minton K . Predicting variant pathogenicity with AlphaMissense. Nat Rev Genet 2023;24:804. 37821682 10.1038/s41576-023-00668-9

[15] Tolosa E , Vila M , Klein C , Rascol O . LRRK2 in Parkinson disease: challenges of clinical trials. Nat Rev Neurol 2020;16:97–107. 31980808 10.1038/s41582-019-0301-2

[16] Golub Y , Berg D , Calne DB , et al. Genetic factors influencing age at onset in LRRK2‐linked Parkinson disease. Parkinsonism Relat Disord 2009;15:539–541. 19041274 10.1016/j.parkreldis.2008.10.008

[17] Gan‐Or Z , Bar‐Shira A , Mirelman A , Gurevich T , Giladi N , Orr‐Urtreger A . The age at motor symptoms onset in LRRK2‐associated Parkinson's disease is affected by a variation in the MAPT locus: a possible interaction. J Mol Neurosci 2012;46:541–544. 21898123 10.1007/s12031-011-9641-0

