# Supplementary material for: Prevalence and Clinical Characteristics of the LRRK2 p.L1795F Variant in Central Europeans with Early‐Onset and Familial Parkinson's Disease
Source: Mov Disord Clin Pract. 2025 Mar 22;12(8):1132–9. doi: 10.1002/mdc3.70045 (PMC12371444; doi:10.1002/mdc3.70045)
Supplement: Supplementary file 1 — Table S1. Primer design and optimised PCR programme used for p.L1795F variant validation. Table S2. Characteristics of the PD patients included in the WES study group. Table S3. Characteristics of the HC included in the study. Table S4. List of PD‐associated genes screened in our PD cohort. Table S5. Identity‐by‐descent (IBD) calculation. Table S6. Additional clinical information of identified LRRK2 p.L1795F positive PD patients. Table S7. The overlapping identify‐by‐descent segments spanning LRRK2 p.L1795F variant among the carriers genotyped by whole‐exome sequence and array. Table S8. The common haplotype (grey) shared by LRRK2 p.L1795F (red) carriers inferred from the whole‐exome‐sequence and array data. Figure S1. B‐allele frequency and Log‐R ratio plots of the LRRK2 p.L1795F positive carriers. Figure S2. p.L1795F variant's validation by Sanger sequencing. Figure S3. (A) CryoEM structure for the LRRK2 dimer with highlighted PD‐associated mutations including the proposed p.L1795F variant (B) proximity of p.L1795F to previously demonstrated pathogenic variants in the ROC and COR domains. Image derived from PDB 7LHT using chimera X. [file MDC3-12-1132-s001.docx]

**Supplementary**

**Title**: Prevalence and clinical characteristics of the LRRK2 p.L1795F variant in Central Europeans with early-onset and familial Parkinson's disease

**Legend:**

[Standardised protocol used to collect clinical data 5](#_Toc192191349)

[Genetic studies 6](#_Toc192191350)

[Supp. Table 1: Primer design and optimised PCR programme used for p.L1795F variant validation 12](#_Toc192191351)

[Supp. Table 2: Characteristics of the PD patients included in the WES study group 14](#_Toc192191352)

[Supp. Table 3: Characteristics of the HC included in the study. 16](#_Toc192191353)

[Supp. Table 4: List of PD-associated genes screened in our PD cohort 17](#_Toc192191354)

[Supp. Table 5: Identity-by-descent (IBD) calculation 19](#_Toc192191355)

[Supp. Table 6: Additional clinical information of identified *LRRK2* p.L1795F positive PD patients. 20](#_Toc192191356)

[Supp. Table 7: The overlapping identify-by-descent segments spanning LRRK2 p.L1795F variant among the carriers genotyped by whole-exome sequence and array 22](#_Toc192191357)

[Supp. Table 8: The common haplotype (grey) shared by LRRK2 p.L1795F (red) carriers inferred from the whole-exome-sequence and array data 24](#_Toc192191358)

[Supp. Figure 1: B-allele frequency and Log-R ratio plots of the *LRRK2* p.L1795F positive carriers 27](#_Toc192191359)

[Supp. Figure 2: p.L1795F variant’s validation by Sanger sequencing 31](#_Toc192191363)

[Supp. Figure 3: (A) CryoEM structure for the LRRK2 dimer with highlighted PD-associated mutations including the proposed p.L1795F variant (B) proximity of p.L1795F to previously demonstrated pathogenic variants in the ROC and COR domains. Image derived from PDB 7LHT using chimera X [^1,2^]. 32](#_Toc192191364)

# **Standardised protocol used to collect clinical data**

The Movement Disorder Society-Unified Parkinson's Disease Rating (MDS-UPDRS) Part III (Goetz et al. 2008) and Hoehn and Yahr (H&Y) stage systems (Hoehn and Yahr 1967) were used to evaluate motor severity. Cognitive functions were evaluated using the Montreal Cognitive Assessment (MoCA) (Nasreddine et al. 2005) and the Parkinson's Disease - Cognitive Rating Scale (PD-CRS) (Pagonabarraga et al. 2008).  Non-Motor Symptoms Scale (NMSS) (Chaudhuri et al. 2007) was used for the assessment of non-motor symptoms in PD and autonomic dysfunction was reported using the Scale for Outcomes in Parkinson's disease for Autonomic symptoms (SCOPA-AUT) (Visser et al. 2004). The REM Sleep Behaviour Disorder Screening Questionnaire (RBDSQ) (Stiasny-Kolster et al. 2007) was used as a self-screening tool for RBD. The general level of daytime sleepiness using The Epworth Sleepiness Scale (ESS) (Johns 1991). Fatigue was measured with the Multidimensional Fatigue Inventory (MFI) (Smets et al. 1995). The Beck Depression Inventory II (BDI-II) (Beck et al. 1996) and the Parkinson Anxiety Scale (PAS) (Leentjens et al. 2014) were used to assess for mood problems. The Impulsive-Compulsive Disorders were reported via the Questionnaire for Impulsive-Compulsive Disorders in Parkinson's Disease - Rating Scale (QUIP-RS) (Weintraub et al. 2012). The 39-Item Parkinson's Disease Questionnaire (PDQ-39) was used to assess PD-specific health-related quality over the last month (Jenkinson et al. 1997).

# **Genetic studies**

Alignment and variant calling in WES data

Three patients (F1-III-1; F2-II-1 and F3-III-6) had WES data available at the time of this study. Raw FASTQ data were aligned against the GRCh38 human reference assembly using the BWA Burrow-Wheeler Aligner. Picard tools were used for marking duplicates (<https://github.com/broadinstitute/picard>). BAM files were generated using SAMtools. (<http://samtools.sourceforge.net/>).  GATK v4.0.4.0; <https://www.broadinstitute.org/gatk/>) was used for base quality score recalibration, variant calling and variant quality score recalibration. Variants were annotated using VEP Variant Effect Predictor(<https://genomebiology.biomedcentral.com/articles/10.1186/s13059-016-0974-4>).  cDNA and protein sequence variants are described in accordance with the recommendations of Human Genome Variation Society. All LRRK2 variants were harmonized with the canonical Ensemble feature ENSG00000188906; ENST00000298910 (RefSeq NM_198578.4) of the GRCh38/hg38 human reference genome build.

Variant filtering

Following alignment, variants were filtered using specific thresholds for several annotations using the custom in-house pipeline. Synonymous and common variants with a population allele frequency (AF) ≥0.05 (5%) reported in the Genome Aggregation Database v3 (gnomAD v3) or Exome Aggregation Consortium (ExAC) database or reported to be common in the in-house WES database as well as variants predicted to be of functionality ‘low impact’ were removed. Missense variants were predicted *in silico* using Sort Intolerant from Tolerant (SIFT) and Polymorphism Phenotyping v2 (PolyPhen2); those labelled to be ‘benign’ or ‘tolerated’ were excluded. Lastly, variants within PD-associated genes based on Genomics England Parkinson Disease and Complex Parkinsonism panel list v1.120 (<https://panelapp.genomicsengland.co.uk/panels/39/>) and published literature (Blauwendraat, Nalls, and Singleton 2020; Gustavsson et al. 2024; Magrinelli et al. 2024) were included (Suppl. Table 3).

Variant validation

Primers for mutation validation were designed using Primer3 software (Rozen and Skaletsky 2000). Reference sequences of the human genome assembly build 38 (GRCh38) were obtained from the Ensemble Genome Browser (<http://www.ensembl.org/index.html>). The Primer-BLAST online tool (https://www.ncbi.nlm.nih.gov/tools/primer-blast/) was used to ensure the designed primers were specific to the target of interest, and the designed primers ordered (Sigma-Aldrich, USA) are stated in Suppl. Table 1. The PCR reaction was then optimised and confirmed through agarose gel electrophoresis (Suppl. Table 8).  The purified PCR product (Exosap, Thermo Fisher Scientific, USA) was then sequenced and read by the ABI 3730xl DNA analyser (Applied Biosystems, USA). The sequencing reads were checked using Sequencer software version 4.1.4.

Genotyping using GSA 24 v.3.0. array

Four patients (Patient F1-III-1; F2-II-1; F3-III-6 and F4-III-2) had the genotyping data from the GSA-24 v.3.0 array available at the time of this study. Briefly, the method used gDNA whole-genome amplification and enzymatic fragmentation to produce pieces around 300 bp in length. The gDNA fragments were subsequently purified and hybridised on Illumina Bead Chips, which are microbeads with SNP-specific oligonucleotide capture probes attached. Allele-specific antibodies labelled with either biotin or DNP were used to identify hybridised fragments, which were then scanned with a two-confocal laser and shown on an Illumina Bead Array reader. The transmitted colour signal was then used to identify the genotype. The genotyping data were then generated in the raw IDATs files that were imported to GenomeStudio 2.0 software and underwent several quality-control procedures (QCs). All samples passed the following QCs steps: all samples had call rate above 0.95 (95%), none of them showed excessive or deficient proportion of heterozygote genotypes suggesting the possibility of gDNA sample contamination or inbreeding and to exclude possible gender discrepancy, the homozygosity rate across all X-chromosome SNPs was calculated and compared the estimated rate with the expected value (F value between 0.25 and 0.75).

**References:**

Beck, A. T., R. A. Steer, R. Ball, and W. Ranieri. 1996. ‘Comparison of Beck Depression Inventories -IA and -II in Psychiatric Outpatients’. *Journal of Personality Assessment* 67 (3): 588–97. https://doi.org/10.1207/s15327752jpa6703_13.

Blauwendraat, Cornelis, Mike A. Nalls, and Andrew B. Singleton. 2020. ‘The Genetic Architecture of Parkinson’s Disease’. *Lancet Neurology* 19 (2): 170–78. https://doi.org/10.1016/S1474-4422(19)30287-X.

Chaudhuri, Kallol Ray, Pablo Martinez-Martin, Richard G. Brown, Kapil Sethi, Fabrizio Stocchi, Per Odin, William Ondo, et al. 2007. ‘The Metric Properties of a Novel Non-Motor Symptoms Scale for Parkinson’s Disease: Results from an International Pilot Study’. *Movement Disorders: Official Journal of the Movement Disorder Society* 22 (13): 1901–11. https://doi.org/10.1002/mds.21596.

Goetz, Christopher G., Barbara C. Tilley, Stephanie R. Shaftman, Glenn T. Stebbins, Stanley Fahn, Pablo Martinez-Martin, Werner Poewe, et al. 2008. ‘Movement Disorder Society-Sponsored Revision of the Unified Parkinson’s Disease Rating Scale (MDS-UPDRS): Scale Presentation and Clinimetric Testing Results’. *Movement Disorders: Official Journal of the Movement Disorder Society* 23 (15): 2129–70. https://doi.org/10.1002/mds.22340.

Gustavsson, Emil K., Jordan Follett, Joanne Trinh, Sandeep K. Barodia, Raquel Real, Zhiyong Liu, Melissa Grant-Peters, et al. 2024. ‘RAB32 Ser71Arg in Autosomal Dominant Parkinson’s Disease: Linkage, Association, and Functional Analyses’. *Lancet Neurology* 23 (6): 603–14. https://doi.org/10.1016/S1474-4422(24)00121-2.

Hoehn, M. M., and M. D. Yahr. 1967. ‘Parkinsonism: Onset, Progression and Mortality’. *Neurology* 17 (5): 427–42. https://doi.org/10.1212/wnl.17.5.427.

Jenkinson, C., R. Fitzpatrick, V. Peto, R. Greenhall, and N. Hyman. 1997. ‘The Parkinson’s Disease Questionnaire (PDQ-39): Development and Validation of a Parkinson’s Disease Summary Index Score’. *Age and Ageing* 26 (5): 353–57. https://doi.org/10.1093/ageing/26.5.353.

Johns, M. W. 1991. ‘A New Method for Measuring Daytime Sleepiness: The Epworth Sleepiness Scale’. *Sleep* 14 (6): 540–45. https://doi.org/10.1093/sleep/14.6.540.

Leentjens, Albert F. G., Kathy Dujardin, Gregory M. Pontone, Sergio E. Starkstein, Daniel Weintraub, and Pablo Martinez-Martin. 2014. ‘The Parkinson Anxiety Scale (PAS): Development and Validation of a New Anxiety Scale’. *Movement Disorders: Official Journal of the Movement Disorder Society* 29 (8): 1035–43. https://doi.org/10.1002/mds.25919.

Magrinelli, Francesca, Christelle Tesson, Plamena R. Angelova, Ainara Salazar-Villacorta, Jose A. Rodriguez, Annarita Scardamaglia, Brian Hon-Yin Chung, et al. 2024. ‘PSMF1 Variants Cause a Phenotypic Spectrum from Early-Onset Parkinson’s Disease to Perinatal Lethality by Disrupting Mitochondrial Pathways’. *MedRxiv: The Preprint Server for Health Sciences*, June. https://doi.org/10.1101/2024.06.19.24308302.

Nasreddine, Ziad S., Natalie A. Phillips, Valérie Bédirian, Simon Charbonneau, Victor Whitehead, Isabelle Collin, Jeffrey L. Cummings, and Howard Chertkow. 2005. ‘The Montreal Cognitive Assessment, MoCA: A Brief Screening Tool for Mild Cognitive Impairment’. *Journal of the American Geriatrics Society* 53 (4): 695–99. https://doi.org/10.1111/j.1532-5415.2005.53221.x.

Pagonabarraga, Javier, Jaime Kulisevsky, Gisela Llebaria, Carmen García-Sánchez, Berta Pascual-Sedano, and Alexandre Gironell. 2008. ‘Parkinson’s Disease-Cognitive Rating Scale: A New Cognitive Scale Specific for Parkinson’s Disease’. *Movement Disorders: Official Journal of the Movement Disorder Society* 23 (7): 998–1005. https://doi.org/10.1002/mds.22007.

Rozen, S., and H. Skaletsky. 2000. ‘Primer3 on the WWW for General Users and for Biologist Programmers’. *Methods in Molecular Biology*  132: 365–86. https://doi.org/10.1385/1-59259-192-2:365.

Smets, E. M., B. Garssen, B. Bonke, and J. C. De Haes. 1995. ‘The Multidimensional Fatigue Inventory (MFI) Psychometric Qualities of an Instrument to Assess Fatigue’. *Journal of Psychosomatic Research* 39 (3): 315–25. https://doi.org/10.1016/0022-3999(94)00125-o.

Stiasny-Kolster, Karin, Geert Mayer, Sylvia Schäfer, Jens Carsten Möller, Monika Heinzel-Gutenbrunner, and Wolfgang H. Oertel. 2007. ‘The REM Sleep Behavior Disorder Screening Questionnaire--a New Diagnostic Instrument’. *Movement Disorders: Official Journal of the Movement Disorder Society* 22 (16): 2386–93. https://doi.org/10.1002/mds.21740.

Visser, Martine, Johan Marinus, Anne M. Stiggelbout, and Jacobus J. Van Hilten. 2004. ‘Assessment of Autonomic Dysfunction in Parkinson’s Disease: The SCOPA-AUT’. *Movement Disorders: Official Journal of the Movement Disorder Society* 19 (11): 1306–12. https://doi.org/10.1002/mds.20153.

Weintraub, Daniel, Eugenia Mamikonyan, Kimberly Papay, Judith A. Shea, Sharon X. Xie, and Andrew Siderowf. 2012. ‘Questionnaire for Impulsive-Compulsive Disorders in Parkinson’s Disease-Rating Scale’. *Movement Disorders: Official Journal of the Movement Disorder Society* 27 (2): 242–47. https://doi.org/10.1002/mds.24023.

# **Supp. Table 1: Primer design and optimised PCR programme used for p.L1795F variant validation**

| **Primer’s sequence (5’-3’)** | | | |
| --- | --- | --- | --- |
| Forward: | TAGACAAGGTGTTGAGCTCT | | |
| Reverse: | CATACCTTCCTCTGCTTTCT | | |
| **Optimized PCR programme** | | | |
| **Step** | **Temperature** | **Time** | **Number of cycles** |
| Denaturation | 94°C | 10 minutes | x1 |
| Denaturation | 94°C | 30 seconds | x8 |
| Annealing | 57°C | 30 seconds |  |
| Elongation | 72°C | 45 seconds |  |
| Denaturation | 94°C | 30 seconds | x16 |
| Annealing | 57°C  (-0.7°C per cycle) | 30 seconds |  |
| Elongation | 72°C | 45 seconds |  |
| Denaturation | 94°C | 30 seconds | x16 |
| Annealing | 47°C | 30 seconds |  |
| Elongation | 72°C | 30 seconds |  |
| Elongation | 72°C | 5 minutes | x1 |
| Hold | 4°C | | |

# **Supp. Table 2: Characteristics of the PD patients included in the WES study group**

|  | **PD patients (n=219)** | | |
| --- | --- | --- | --- |
|  | **Mean±SD (n, %)** | **Median** | **Range** |
| **Age** | 53.5±12.9 | 51 | 17-83 |
| **Gender** |  | | |
| **Male** | 136 (62%) | | |
| **Female** | 83 (38%) | | |
| **Ethnicity** |  | | |
| **Czech** | 56 (26%) | | |
| **Hungarian** | 43 (20%) | | |
| **Polish** | 19 (9%) | | |
| **Slovak** | 101 (46%) | | |
| **Age at onset** | 41.7±11.2 | 39 | 14-80 |
| **< 40 years** | 117 (53%) | | |
| **< 50 years** | 179(82%) | | |
| **Disease duration** | 12±8.9 | 10 | 1-45 |
| **Positive family history** | 93 (43%) | | |
| **1st degree relative with PD** | 58 (26.5%) | | |
| **2nd degree relative with PD** | 33 (15.1%) | | |
| **Advanced therapy** | | | |
| **Apomorphine pump** | 2 (1%) | | |
| **LCIG /Subcutaneous Levodopa-Carbidopa** | 16 (7.9%) | | |
| **DBS** | 77 (38%) | | |

PD = Parkinson’s Disease; LCIG = Levodopa/Carbidopa Intestinal Gel; DBS = Deep Brain Stimulation;SD = standard deviation

# **Supp. Table 3: Characteristics of the HC included in the study.**

|  | **HC (n = 303)** | | |
| --- | --- | --- | --- |
|  | **Mean±SD (n, %)** | **Median** | **Range** |
| **Age** | 62.93±13.51 | 64 | 30-102 |
| **Gender** |  | | |
| **Male** | 129 (42.6%) | | |
| **Female** | 174 (57.4%) | | |
| **Ethnicity** |  | | |
| **Hungarian – region bordering Slovakia** | 195 (64.4%) | | |
| **Slovak – eastern region** | 108 (35.6%) | | |

HC = healthy controls; SD = standard deviation

# **Supp. Table 4: List of PD-associated genes screened in our PD cohort**

| **PD WES study cohort (n= 219)** | |
| --- | --- |
| **Gene** | **Reference** |
| *ATP13A2, ATP1A3, C19orf12, CSF1R, DCTN1, DNAJC6, FBXO7, FTL, GBA, GCH1, GRN, LRRK2, LYST, MAPT, OPA3, PANK2, PARK7, PDGFB, PINK1, PLA2G6, PRKN, PRKRA, PTRHD1, RAB39B, SLC30A10, SLC39A14, SLC6A3, SNCA, SPG11, SPR, SYNJ1, TH, TUBB4A, VPS13A, VPS35, WDR45* | Genomics England Parkinson Disease and Complex Parkinsonism panel green list v1.120 |
| *CHCHD2, COASY, TAF1* | Genomics England Parkinson Disease and Complex Parkinsonism panel amber list v1.120 |
| *ANO3, ATP6AP2, EIF4G1, GIGYF2, GNAL, HTRA2, IPPK, NR4A2, SGCE, SLC4A1, SNCAIP, THAP1, TOR1A, UCHL1* | Genomics England Parkinson Disease and Complex Parkinsonism panel red list v1.120 |
| *POLG, DNAJC13, TMEM230, VPS13C, LRP10* | Blauwendrat et al, 2020 ^1^ |
| *RAB32* | Gustavsson et al., 2024 ^2^ |
| *PMSF1* | Magrinelli et al.2024 ^3^ |
| **Patient *F2-II-1*** | |
| *PD gene panel: PARK2, PINK1, PARK7, LRRK2 including screening the CNVs with the MLPA* | A previous local genetic report conducted in 2018 |

PD = Parkinson’s Disease; WES = Whole Exome Sequencing; CNVs = Copy Number Variations; MLPA = Multiplex Ligation-Dependent Probe Amplification

^1^ Blauwendraat C et al. The genetic architecture of Parkinson's disease. Lancet Neurol. 2020;19(2):170-178; ^2^ Gustavsson, Emil K et al. RAB32 Ser71Arg in autosomal dominant Parkinson's disease: linkage, association, and functional analyses Lancet Neurol. 2024; 23(6):603 – 614; ^3^ Magrinelli F et al. PSMF1 variants cause a phenotypic spectrum from early-onset Parkinson's disease to perinatal lethality by disrupting mitochondrial pathways. medRxiv [Preprint], 2024; 20:2024.06.19.24308302.

# **Supp. Table 5: Identity-by-descent (IBD) calculation**

| **ID1** | **ID2** | **Z0** | **Z1** | **Z2** | **PI_HAT** | **PHE** | **DST** | **PPC** | **RATIO** |
| --- | --- | --- | --- | --- | --- | --- | --- | --- | --- |
| ***F4-III-2*** | ***F2-II-1*** | 1 | 0 | 0 | 0 | 1 | 0.731923 | 0.6246 | 2.021 |
| ***F1-III-1*** | ***F2-II-1*** | 0.977 | 0.0179 | 0.0051 | 0.0141 | 1 | 0.735214 | 0.9962 | 2.1858 |
| ***F3-III-6*** | ***F2-II-1*** | 1 | 0 | 0 | 0 | 1 | 0.731249 | 0.0806 | 1.9107 |
| ***F4-III-2*** | ***F3-III-6*** | 1 | 0 | 0 | 0 | 1 | 0.731102 | 0.1905 | 1.9435 |
| ***F3-III-6*** | ***F1-III-1*** | 1 | 0 | 0 | 0 | 1 | 0.731643 | 0.0966 | 1.917 |
| ***F4-III-2*** | ***F1-III-1*** | 0.9889 | 0.0111 | 0 | 0.0056 | 1 | 0.732428 | 0.5992 | 2.0165 |

ID1 = individual 1; ID2 = individual 2; Z0 = P(IBD=0); Z1 = P(IBD=1); Z2 = P(IBD=2); PI_HAT = Proportion IBD; PHE = pairwise phenotypic code; DST = IBS distance; PPC = IBS binomial test; RATIO = HETHET : IBS0 SNP ratio (expected value 2);

# **Supp. Table 6: Additional clinical information of identified *LRRK2* p.L1795F positive PD patients.**

|  | ***F1-III-1*** | ***F1-III-2*** | ***F2-II-1*** | ***F3-III-5*** | ***F3-III-6*** | ***F4-III-2*** |
| --- | --- | --- | --- | --- | --- | --- |
| **NMSS score** | 18 | 29 | 69 | NA | 57 | NA |
| **RBDSQ score** | 3 | 2 | 5 | NA | 5 | 1 |
| **PDQ-39 score** | 4.6 | 5.1 | 31.3 | 36.4 | 44.6 | 38.9 |
| **QUIP-RS score** | 0 | 1 | 7 | NA | 0 | NA |
| **BDI-II score** | 12 | 22 | 25 | 17 | 33 | 22 |
| **PDSS-2 score** | 19 | 5 | 21 | 27 | 15 | 37 |
| **SCOPA-AUT score** | 14 | 3 | 26 | 14 | 6 | 18 |
| **AS score** | 8 | 7 | 21 | NA | 1 | NA |
| **PD-CRS**  **Cortical score**  **Subcortical score**  **Total score** | 30    84    114 | 30  57  87 | 28    42    70 | NA  NA  NA | NA    NA    NA | 29    50    79 |
| **ESS score** | 12 | 6 | 2 | 11 | 5 | 3 |
| **MFI general fatigue** | 10 | 12 | NA | NA | NA | 4 |
| **MFI physical fatigue** | 6 | 13 | NA | NA | NA | 6 |
| **MFI reduced activity** | 8 | 11 | NA | NA | NA | 10 |
| **MFI reduced motivation** | 13 | 12 | NA | NA | NA | 15 |
| **MFI mental fatigue** | 16 | 14 | NA | NA | NA | 15 |
| **Apathy scale score** | 17 | 31 | 10 | 30 | 21 | 11 |

SD = standard deviation; NMSS = Non-Motor Symptoms Scale; RBDSQ = REM Sleep Behaviour Disorder Screening Questionnaire; PDQ-39 = The 39-Item Parkinson's Disease Questionnaire; QUIP-RS = Questionnaire for Impulsive-Compulsive Disorders in Parkinson's Disease - Rating Scale; BDI-II = Beck Depression Inventory II, PDSS-2 = Parkinson’s Disease Sleep Scale-2; SCOPA-AUT = The Scale for Outcomes in Parkinson's disease for Autonomic symptoms; PAS = The Parkinson Anxiety Scale; PD-CRS = The Parkinson's Disease - Cognitive Rating Scale; ESS = The Epworth Sleepiness Scale; MFI = Multidimensional Fatigue Inventory; FSS = Fatigue severity scale; NA = not available;

# **Supp. Table 7: The overlapping identify-by-descent segments spanning LRRK2 p.L1795F variant among the carriers genotyped by whole-exome sequence and array**

| **Hap-IBD WES** | | | | | |
| --- | --- | --- | --- | --- | --- |
| Sample ID 1 | Sample ID 2 | Chrom | Start | End | cM |
| F3-III-6 | F4-III-2 | 12 | 27969960 | 48348181 | 13.87 |
| F3-III-6 | F1-III-1 | 12 | 29489609 | 46776834 | 10.07 |
| F4-III-2 | F1-III-1 | 12 | 29489609 | 46776834 | 10.07 |
| **Hap-IBD Array** | | | | | |
| Sample ID 1 | Sample ID 2 | Chrom | Start | End | cM |
| F3-III-6 | F4-III-2 | 12 | 30881021 | 48711736 | 10.45 |
| F3-III-6 | F1-III-1 | 12 | 30881021 | 47075948 | 8.49 |
| F4-III-2 | F1-III-1 | 12 | 26805390 | 47075948 | 13.44 |
| F2-II-1 | F3-III-6 | 12 | 32494824 | 40794409 | 2.15 |
| F2-II-1 | F4-III-2 | 12 | 32494824 | 40794409 | 2.15 |
| F2-II-1 | F1-III-1 | 12 | 32494824 | 40794409 | 2.15 |
| **Germline2 WES** | | | | | |
| Sample 1 ID | Sample 2 ID | Chrom | Start | End | cM |
| F3-III-6 | F4-III-2 | 12 | 29659938 | 48132078 | 11.53 |
| F3-III-6 | F1-III-1 | 12 | 29659938 | 46766704 | 9.57 |
| F4-III-2 | F1-III-1 | 12 | 29659938 | 46766704 | 9.57 |
| **Germline2 Array** | | | | | |
| Sample 1 ID | Sample 2 ID | Chrom | Start | End | cM |
| F3-III-6 | F4-III-2 | 12 | 30910494 | 48351335 | 10.15 |
| F3-III-6 | F1-III-1 | 12 | 30910494 | 46774054 | 8.16 |
| F4-III-2 hap1 | F1-III-1 hap2 | 12 | 26990575 | 46774054 | 12.45 |
| F4-III-2 hap1 | F1-III-1 hap1 | 12 | 40303632 | 40495144 | 0.12 |
| F2-II-1 | F3-III-6 | 12 | 32593572 | 40775226 | 1.81 |
| F2-II-1 | F4-III-2 | 12 | 32593572 | 40775226 | 1.81 |
| F2-II-1 | F1-III-1 | 12 | 32593572 | 40775226 | 1.81 |

WES = whole-exome sequencing; Chrom = chromosome, cM = centimorgan; hap = haplotype;

| **Supp. Table 8: The common haplotype (grey) shared by LRRK2 p.L1795F (red) carriers inferred from the whole-exome-sequence and array data**  \|  \| WES \| \| \| \| \| \| Array \| \| \| \| \| \| \| \| **SNP** \| **gnomAD_**  **genome_**  **NFE** \| \| --- \| --- \| --- \| --- \| --- \| --- \| --- \| --- \| --- \| --- \| --- \| --- \| --- \| --- \| --- \| --- \| --- \| \| **SNP ID Chrom:Ref:Alt** \| **F3-III-6_hap1** \| **F4-III-2_hap1** \| **F1-III-1_hap1** \| **F3-III-6_hap2** \| **F4-III-2_hap2** \| **F1-III-1_hap2** \| **F3-III-6_hap1** \| **F4-III-2_hap1** \| **F1-III-1_hap1** \| **F2-II-1_hap1** \| **F3-III-6_hap2** \| **F4-III-2_hap2** \| **F1-III-1_hap2** \| **F2-II-1_hap2** \| \| chr12:40303632:G:A \| . \| . \| . \| . \| . \| . \| A \| A \| A \| A \| A \| G \| G \| G \| rs10784498 \| 0.3525 \| \| chr12:40303859:T:A \| T \| T \| T \| T \| T \| T \| . \| . \| . \| . \| . \| . \| . \| . \| rs72546323 \| 0.0008 \| \| chr12:40304141:C:T \| C \| C \| C \| C \| C \| C \| . \| . \| . \| . \| . \| . \| . \| . \| rs41286480 \| 0.0016 \| \| chr12:40308564:C:A \| C \| C \| C \| C \| C \| C \| . \| . \| . \| . \| . \| . \| . \| . \| rs200526782 \| 0.0000667 \| \| chr12:40308618:A:G \| A \| A \| A \| A \| A \| A \| A \| A \| A \| A \| A \| A \| A \| A \| rs17466213 \| 0.0009 \| \| chr12:40309109:G:A \| . \| . \| . \| . \| . \| . \| G \| G \| G \| G \| G \| G \| G \| G \| rs7133914 \| 0.0681 \| \| chr12:40309124:G:T \| G \| G \| G \| G \| G \| G \| . \| . \| . \| . \| . \| . \| . \| . \| . \| . \| \| chr12:40309145:C:T \| C \| C \| C \| C \| C \| C \| . \| . \| . \| . \| . \| . \| . \| . \| rs72546327 \| 0.0000666 \| \| chr12:40309185:G:A \| G \| G \| G \| G \| G \| G \| G \| G \| G \| G \| G \| G \| G \| G \| rs11175964 \| 0.0673 \| \| chr12:40309226:A:G \| A \| A \| A \| A \| A \| A \| . \| . \| . \| . \| . \| . \| . \| . \| . \| . \| \| chr12:40314015:A:G \| A \| A \| A \| A \| A \| A \| . \| . \| . \| . \| . \| . \| . \| . \| . \| 0.0000668 \| \| chr12:40314059:C:T \| T \| C \| C \| C \| C \| C \| T \| C \| C \| C \| C \| C \| C \| C \| rs33958906 \| 0.0503 \| \| chr12:40314062:A:G \| A \| A \| A \| A \| A \| A \| . \| . \| . \| . \| . \| . \| . \| . \| rs201540075 \| . \| \| chr12:40315309:C:A \| C \| C \| C \| C \| C \| C \| . \| . \| . \| . \| . \| . \| . \| . \| rs113735323 \| 0 \| \| chr12:40315383:G:A \| A \| A \| A \| G \| G \| G \| . \| . \| . \| . \| . \| . \| . \| . \| . \| . \| \| chr12:40320032:C:A \| A \| C \| C \| A \| A \| A \| A \| C \| C \| A \| A \| A \| A \| A \| rs1427263 \| 0.6787 \| \| chr12:40320071:G:A \| . \| . \| . \| . \| . \| . \| A \| A \| A \| G \| G \| G \| G \| G \| . \| . \| \| chr12:40320097:T:C \| . \| . \| . \| . \| . \| . \| T \| T \| T \| T \| T \| T \| T \| T \| rs35303786 \| 0.0126 \| \| chr12:40320099:T:A \| . \| . \| . \| . \| . \| . \| T \| T \| T \| A \| T \| T \| T \| T \| rs11564148 \| 0.3254 \| \| chr12:40320983:T:A \| A \| A \| A \| T \| T \| T \| . \| . \| . \| . \| . \| . \| . \| . \| . \| . \| \| chr12:40322315:G:A \| G \| G \| G \| G \| G \| G \| . \| . \| . \| . \| . \| . \| . \| . \| rs371793739 \| . \| \| chr12:40322386:G:T (LRRK2 p.L1795F) \| **T** \| **T** \| **T** \| **G** \| **G** \| **G** \| **.** \| **.** \| **.** \| **.** \| **.** \| **.** \| **.** \| **.** \| **rs111910483** \| **.** \| \| chr12:40322458:C:T \| T \| T \| T \| C \| C \| C \| T \| T \| T \| C \| C \| C \| C \| C \| . \| . \| \| chr12:40327357:T:C \| . \| . \| . \| . \| . \| . \| T \| T \| T \| T \| T \| T \| T \| T \| rs17444103 \| 0.0175 \| \| chr12:40333343:C:T \| . \| . \| . \| . \| . \| . \| C \| C \| C \| C \| C \| C \| C \| C \| rs28365229 \| 0.0203 \| \| chr12:40335008:A:T \| A \| A \| A \| A \| A \| A \| . \| . \| . \| . \| . \| . \| . \| . \| rs139746572 \| 0.0005 \| \| chr12:40335191:T:C \| T \| T \| T \| T \| T \| T \| . \| . \| . \| . \| . \| . \| . \| . \| rs17444152 \| 0.0055 \| \| chr12:40335205:C:T \| T \| C \| C \| C \| C \| C \| . \| . \| . \| . \| . \| . \| . \| . \| rs2404834 \| 0.1468 \| \| chr12:40337211:A:G \| . \| . \| . \| . \| . \| . \| A \| A \| A \| A \| A \| A \| A \| A \| rs10506154 \| 0.0205 \| \| chr12:40340400:G:A \| . \| . \| . \| . \| . \| . \| G \| G \| G \| G \| G \| G \| G \| G \| rs34637584 \| 0.0003 \| \| chr12:40341862:A:G \| . \| . \| . \| . \| . \| . \| A \| A \| A \| A \| A \| A \| A \| A \| rs17491536 \| 0.0627 \| \| chr12:40343988:T:C \| . \| . \| . \| . \| . \| . \| T \| T \| T \| T \| T \| T \| T \| T \| rs78044369 \| 0.011 \| \| chr12:40346706:G:C \| G \| G \| G \| G \| G \| G \| . \| . \| . \| . \| . \| . \| . \| . \| . \| . \| \| chr12:40346884:A:G \| A \| A \| A \| A \| A \| A \| A \| A \| A \| A \| A \| A \| A \| A \| rs33995883 \| 0.0175 \| \| chr12:40348019:G:A \| . \| . \| . \| . \| . \| . \| G \| A \| A \| G \| G \| G \| G \| G \| . \| . \| \| chr12:40348452:G:A \| G \| G \| G \| G \| G \| G \| G \| G \| G \| A \| G \| G \| G \| G \| rs10878405 \| 0.3455 \| \| chr12:40350973:A:C \| . \| . \| . \| . \| . \| . \| A \| A \| A \| A \| A \| A \| A \| A \| rs7303525 \| 0.1774 \| \| chr12:40351006:G:A \| . \| . \| . \| . \| . \| . \| A \| G \| G \| G \| G \| G \| G \| G \| rs7132187 \| 0.3244 \| \| chr12:40351502:C:T \| C \| C \| C \| C \| C \| C \| . \| . \| . \| . \| . \| . \| . \| . \| rs79805220 \| 0 \| \| chr12:40351723:A:G \| A \| A \| A \| A \| A \| A \| . \| . \| . \| . \| . \| . \| . \| . \| rs35658131 \| 0.0003 \| \| chr12:40351777:T:C \| T \| T \| T \| T \| T \| T \| . \| . \| . \| . \| . \| . \| . \| . \| rs75415619 \| 0 \| \| chr12:40356393:A:C \| . \| . \| . \| . \| . \| . \| A \| A \| A \| A \| A \| A \| A \| A \| rs11564266 \| 0.0205 \| \| chr12:40357572:C:T \| . \| . \| . \| . \| . \| . \| C \| C \| C \| C \| C \| C \| C \| C \| rs80172174 \| 0.0205 \| \| chr12:40357587:G:A \| . \| . \| . \| . \| . \| . \| G \| G \| G \| G \| G \| G \| G \| G \| rs140057135 \| 0 \| \| chr12:40359345:C:T \| C \| C \| C \| C \| C \| C \| . \| . \| . \| . \| . \| . \| . \| . \| rs200002022 \| 0.0001 \| \| chr12:40360506:T:C \| . \| . \| . \| . \| . \| . \| T \| T \| T \| T \| T \| T \| T \| T \| rs11564146 \| 0.0211 \| \| chr12:40360753:C:T \| . \| . \| . \| . \| . \| . \| C \| C \| C \| C \| C \| C \| C \| C \| rs17466570 \| 0.0613 \| \| chr12:40362670:A:C \| . \| . \| . \| . \| . \| . \| A \| C \| C \| A \| A \| A \| A \| A \| . \| . \| \| chr12:40363528:A:G \| G \| A \| A \| A \| A \| A \| G \| A \| A \| A \| A \| A \| A \| A \| rs33962975 \| 0.1484 \|   SNP = single nucleotide polymorphism; Chrom = chromosome; Ref = reference; Alt = alternative; hap1 = haplotype 1; hap2 = haplotype 2; WES = whole exome sequencing; gnomAD NFE = Allele frequency of non-Finnish European population from the Genome Aggregation Database (gnomAD); NA = not available; |
| --- | --- | --- | --- | --- | --- | --- | --- | --- | --- | --- | --- | --- | --- | --- | --- | --- | --- | --- | --- | --- | --- | --- | --- | --- | --- | --- | --- | --- | --- | --- | --- | --- | --- | --- | --- | --- | --- | --- | --- | --- | --- | --- | --- | --- | --- | --- | --- | --- | --- | --- | --- | --- | --- | --- | --- | --- | --- | --- | --- | --- | --- | --- | --- | --- | --- | --- | --- | --- | --- | --- | --- | --- | --- | --- | --- | --- | --- | --- | --- | --- | --- | --- | --- | --- | --- | --- | --- | --- | --- | --- | --- | --- | --- | --- | --- | --- | --- | --- | --- | --- | --- | --- | --- | --- | --- | --- | --- | --- | --- | --- | --- | --- | --- | --- | --- | --- | --- | --- | --- | --- | --- | --- | --- | --- | --- | --- | --- | --- | --- | --- | --- | --- | --- | --- | --- | --- | --- | --- | --- | --- | --- | --- | --- | --- | --- | --- | --- | --- | --- | --- | --- | --- | --- | --- | --- | --- | --- | --- | --- | --- | --- | --- | --- | --- | --- | --- | --- | --- | --- | --- | --- | --- | --- | --- | --- | --- | --- | --- | --- | --- | --- | --- | --- | --- | --- | --- | --- | --- | --- | --- | --- | --- | --- | --- | --- | --- | --- | --- | --- | --- | --- | --- | --- | --- | --- | --- | --- | --- | --- | --- | --- | --- | --- | --- | --- | --- | --- | --- | --- | --- | --- | --- | --- | --- | --- | --- | --- | --- | --- | --- | --- | --- | --- | --- | --- | --- | --- | --- | --- | --- | --- | --- | --- | --- | --- | --- | --- | --- | --- | --- | --- | --- | --- | --- | --- | --- | --- | --- | --- | --- | --- | --- | --- | --- | --- | --- | --- | --- | --- | --- | --- | --- | --- | --- | --- | --- | --- | --- | --- | --- | --- | --- | --- | --- | --- | --- | --- | --- | --- | --- | --- | --- | --- | --- | --- | --- | --- | --- | --- | --- | --- | --- | --- | --- | --- | --- | --- | --- | --- | --- | --- | --- | --- | --- | --- | --- | --- | --- | --- | --- | --- | --- | --- | --- | --- | --- | --- | --- | --- | --- | --- | --- | --- | --- | --- | --- | --- | --- | --- | --- | --- | --- | --- | --- | --- | --- | --- | --- | --- | --- | --- | --- | --- | --- | --- | --- | --- | --- | --- | --- | --- | --- | --- | --- | --- | --- | --- | --- | --- | --- | --- | --- | --- | --- | --- | --- | --- | --- | --- | --- | --- | --- | --- | --- | --- | --- | --- | --- | --- | --- | --- | --- | --- | --- | --- | --- | --- | --- | --- | --- | --- | --- | --- | --- | --- | --- | --- | --- | --- | --- | --- | --- | --- | --- | --- | --- | --- | --- | --- | --- | --- | --- | --- | --- | --- | --- | --- | --- | --- | --- | --- | --- | --- | --- | --- | --- | --- | --- | --- | --- | --- | --- | --- | --- | --- | --- | --- | --- | --- | --- | --- | --- | --- | --- | --- | --- | --- | --- | --- | --- | --- | --- | --- | --- | --- | --- | --- | --- | --- | --- | --- | --- | --- | --- | --- | --- | --- | --- | --- | --- | --- | --- | --- | --- | --- | --- | --- | --- | --- | --- | --- | --- | --- | --- | --- | --- | --- | --- | --- | --- | --- | --- | --- | --- | --- | --- | --- | --- | --- | --- | --- | --- | --- | --- | --- | --- | --- | --- | --- | --- | --- | --- | --- | --- | --- | --- | --- | --- | --- | --- | --- | --- | --- | --- | --- | --- | --- | --- | --- | --- | --- | --- | --- | --- | --- | --- | --- | --- | --- | --- | --- | --- | --- | --- | --- | --- | --- | --- | --- | --- | --- | --- | --- | --- | --- | --- | --- | --- | --- | --- | --- | --- | --- | --- | --- | --- | --- | --- | --- | --- | --- | --- | --- | --- | --- | --- | --- | --- | --- | --- | --- | --- | --- | --- | --- | --- | --- | --- | --- | --- | --- | --- | --- | --- | --- | --- | --- | --- | --- | --- | --- | --- | --- | --- | --- | --- | --- | --- | --- | --- | --- | --- | --- | --- | --- | --- | --- | --- | --- | --- | --- | --- | --- | --- | --- | --- | --- | --- | --- | --- | --- | --- | --- | --- | --- | --- | --- | --- | --- | --- | --- | --- | --- | --- | --- | --- | --- | --- | --- | --- | --- | --- | --- | --- | --- | --- | --- | --- | --- | --- | --- | --- | --- | --- | --- | --- | --- | --- | --- | --- | --- | --- | --- | --- | --- | --- | --- | --- | --- | --- | --- | --- | --- | --- | --- | --- | --- | --- | --- | --- | --- | --- | --- | --- | --- | --- | --- | --- | --- | --- | --- | --- | --- | --- | --- | --- | --- | --- | --- | --- | --- | --- | --- | --- | --- | --- | --- | --- | --- | --- | --- | --- | --- | --- | --- | --- | --- | --- | --- | --- | --- | --- | --- | --- | --- | --- | --- | --- | --- | --- | --- | --- | --- | --- | --- | --- | --- | --- | --- | --- | --- | --- | --- | --- | --- | --- | --- | --- | --- | --- | --- | --- | --- | --- | --- | --- | --- | --- | --- | --- | --- | --- | --- | --- | --- | --- | --- | --- | --- | --- | --- | --- | --- | --- | --- | --- | --- | --- | --- | --- | --- | --- | --- | --- | --- | --- | --- | --- | --- | --- | --- | --- | --- | --- | --- | --- | --- | --- | --- | --- | --- | --- | --- | --- | --- | --- | --- | --- | --- | --- | --- | --- | --- | --- | --- | --- | --- | --- | --- | --- | --- | --- | --- | --- | --- | --- | --- | --- | --- | --- | --- | --- | --- | --- | --- | --- | --- | --- | --- | --- | --- | --- | --- | --- | --- |

# **Supp. Figure 1: B-allele frequency and Log-R ratio plots of the *LRRK2* p.L1795F positive carriers**


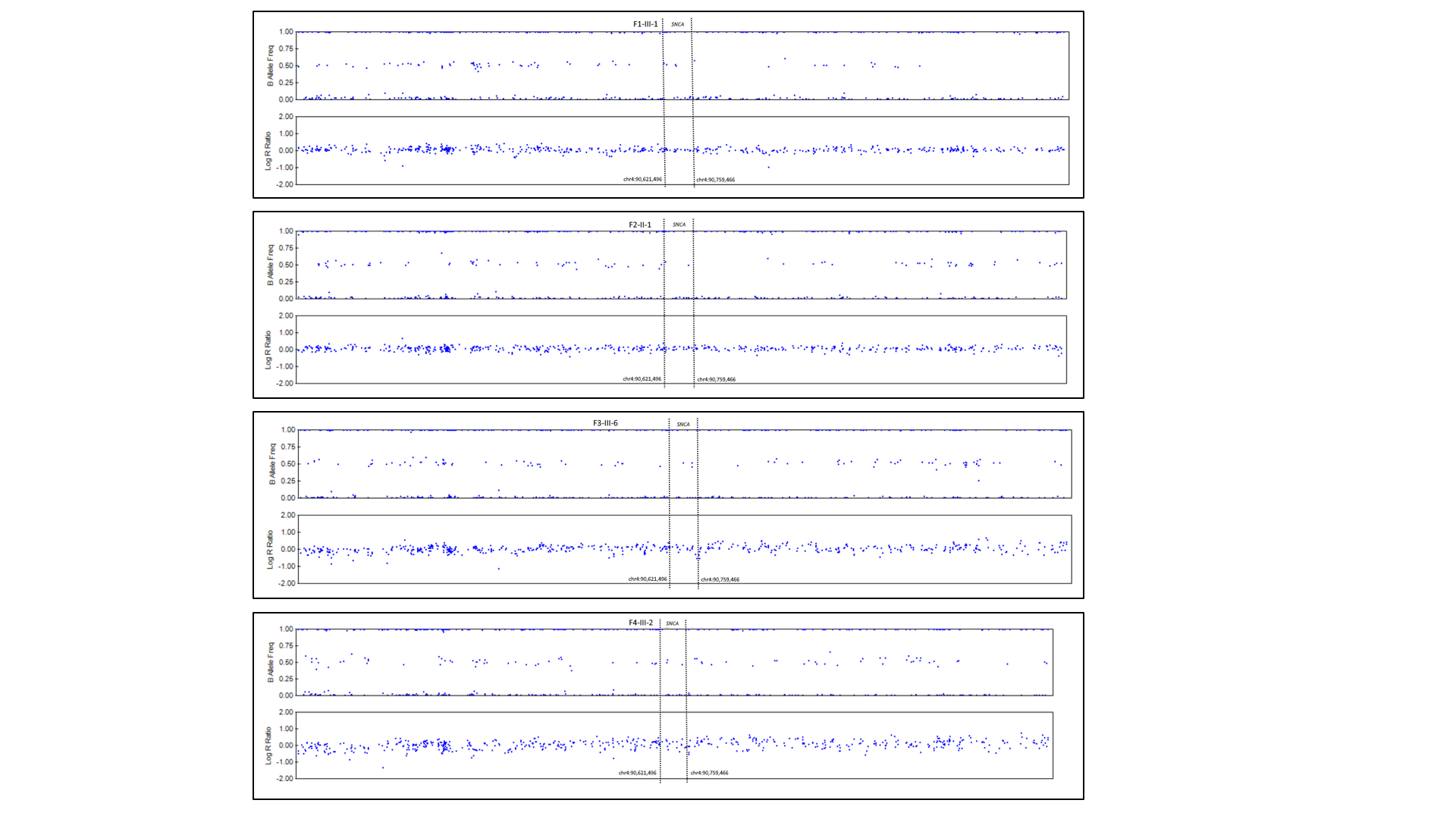


**
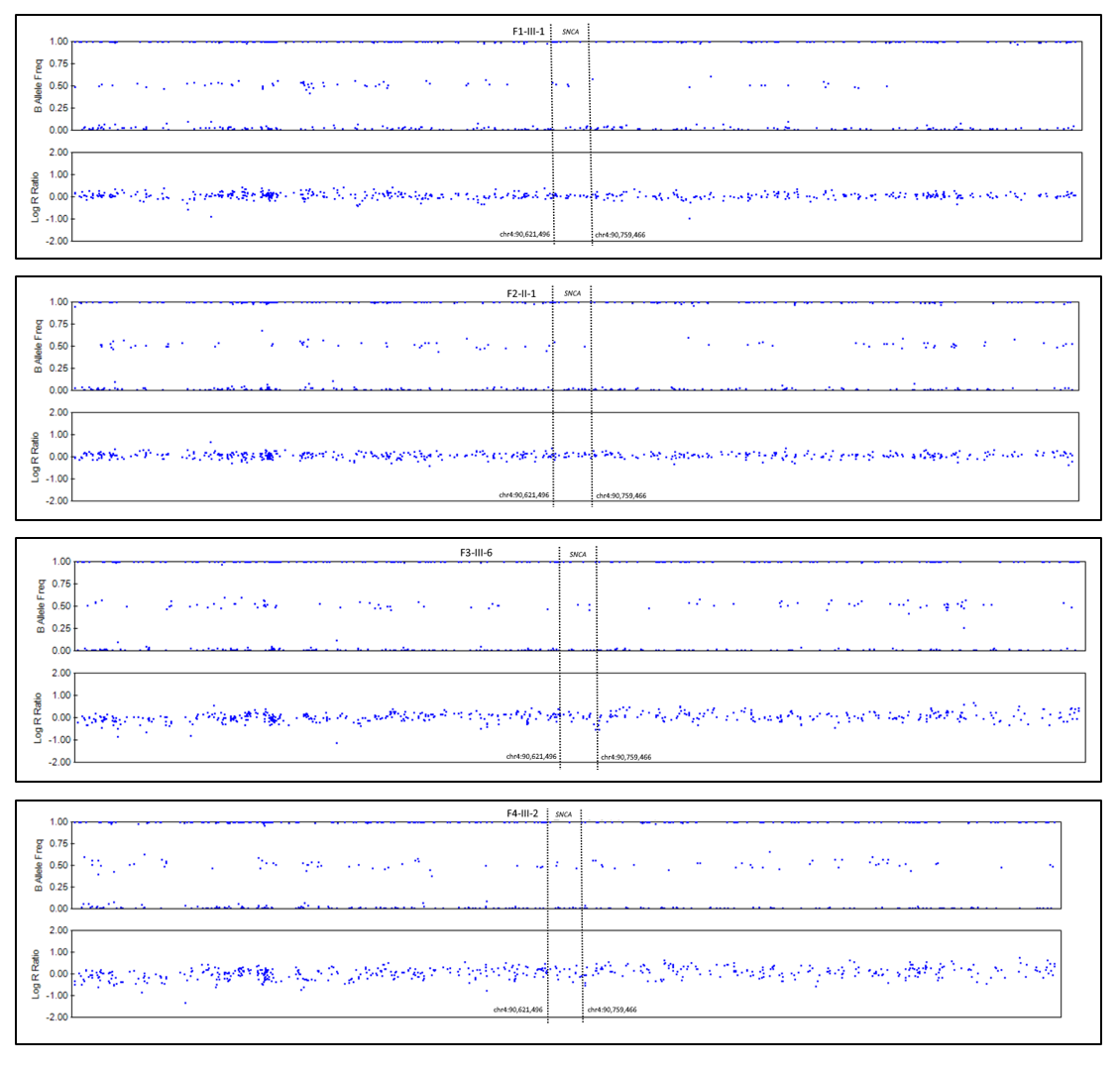
**

**
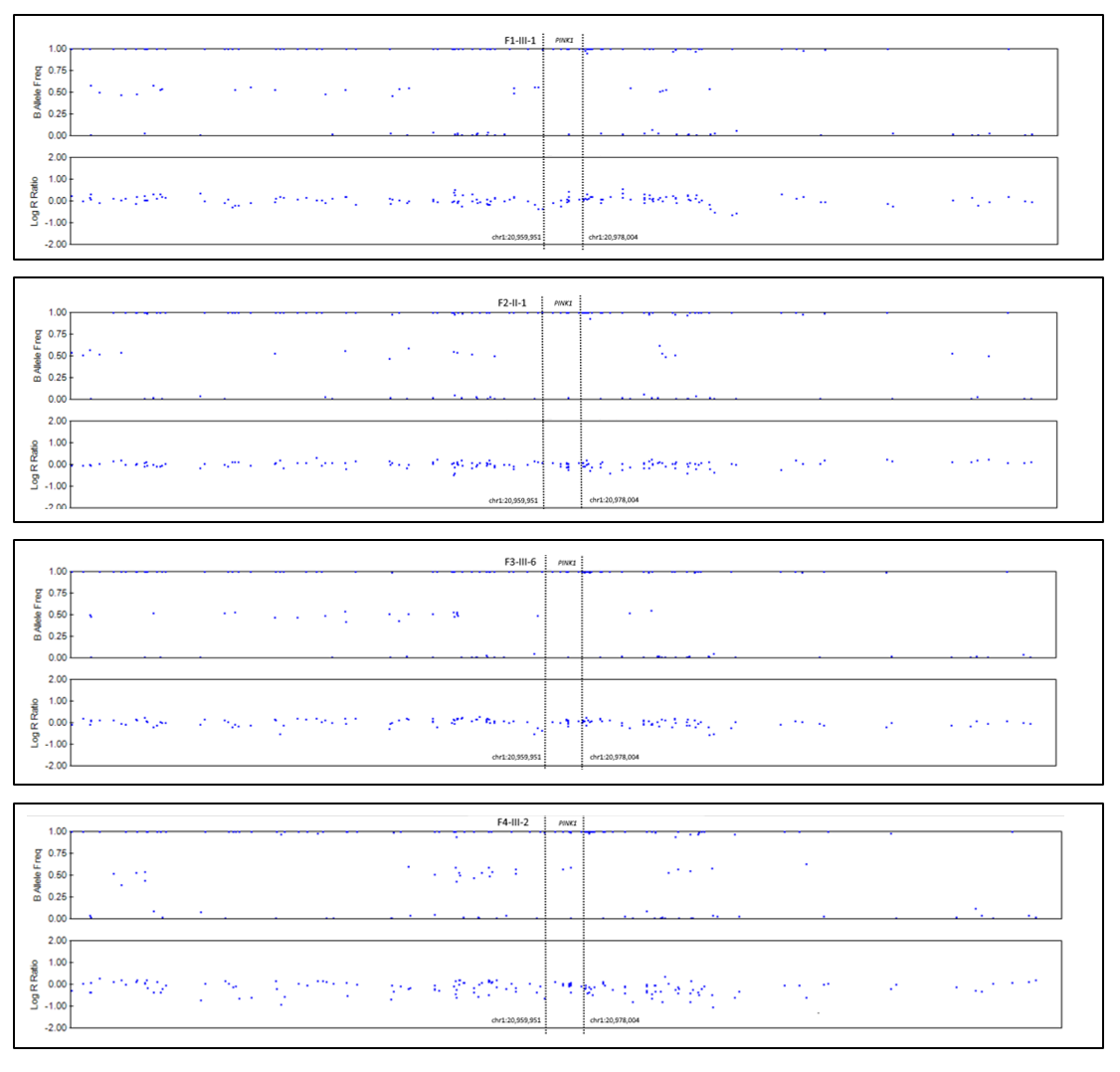
**

**
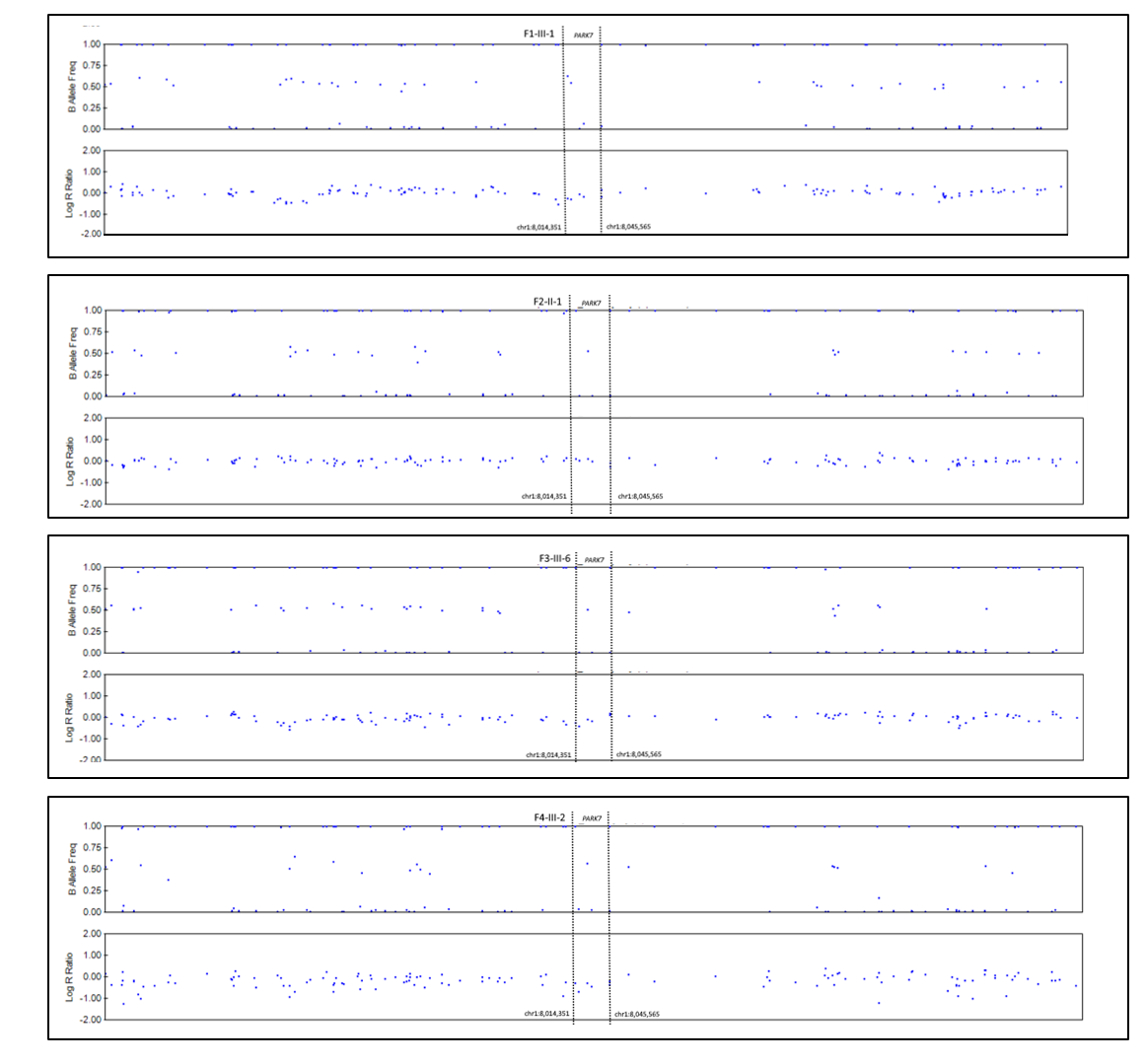
**

# **Supp. Figure 2: p.L1795F variant’s validation by Sanger sequencing**


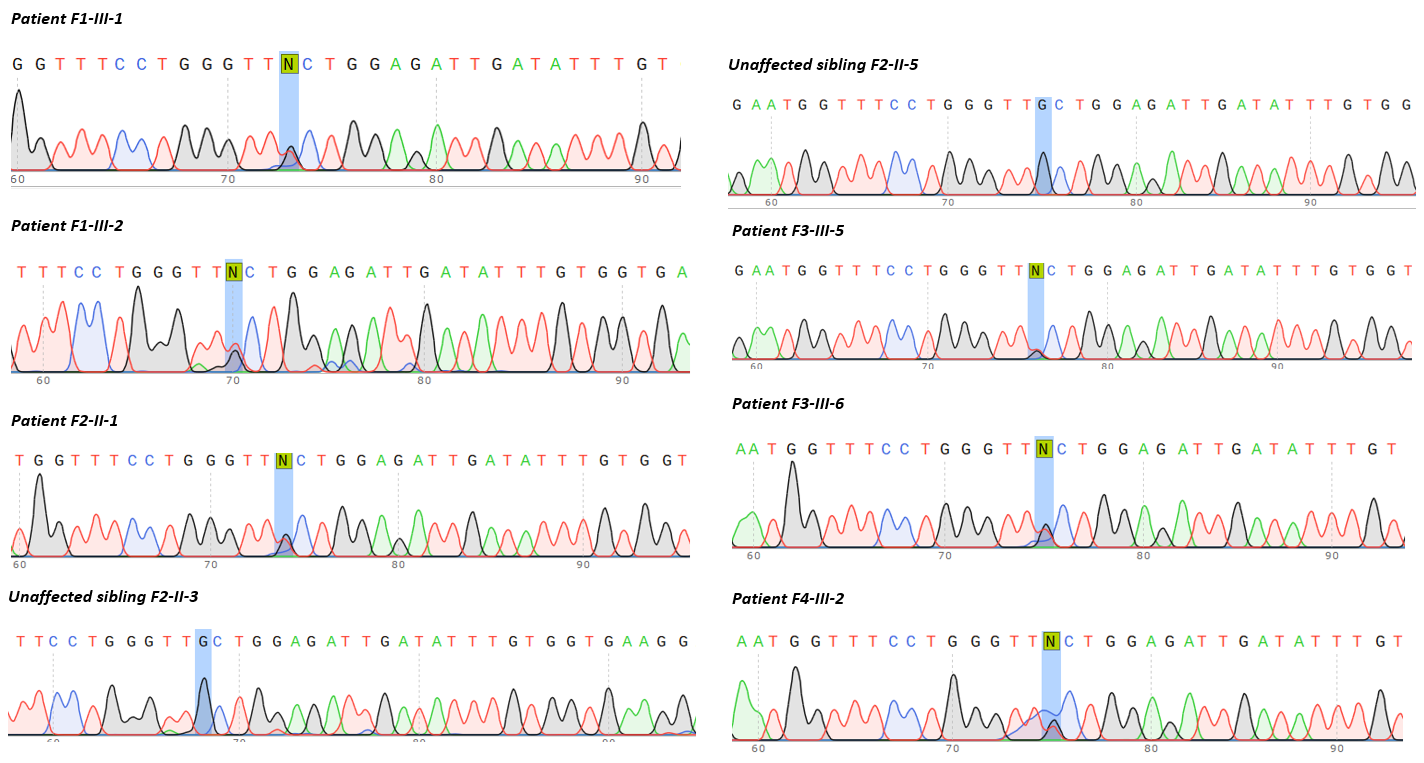


Chromatograph with the *LRRK2* p.L1795F variant as [N].

# **Supp. Figure 3**: (A) CryoEM structure for the LRRK2 dimer with highlighted PD-associated mutations including the proposed p.L1795F variant (B) proximity of p.L1795F to previously demonstrated pathogenic variants in the ROC and COR domains. Image derived from PDB 7LHT using chimera X [^1,2^].

**
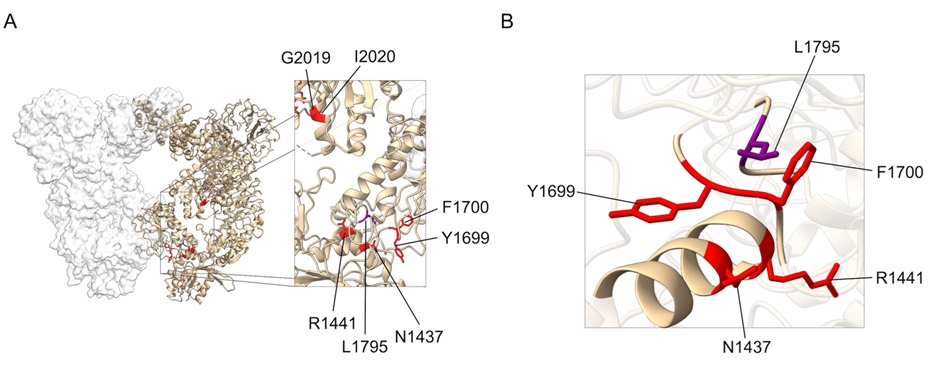
**

1. Myasnikov, A. *et al.* Structural analysis of the full-length human LRRK2. *Cell* **184**, 3519-3527.e10 (2021).

2. Meng, E. C. *et al.* UCSF ChimeraX: Tools for structure building and analysis. *Protein Sci.* **32**, e4792 (2023).
